# The Outcomes of Delayed Revascularization in Lower Extremity Vascular Injury: A Retrospective Cohort Study

**DOI:** 10.5704/MOJ.2503.008

**Published:** 2025-03

**Authors:** KS Chai, WI Faisham, WA Wan-Sulaiman, MA Rosli, AZ Mat-Saad, MH Jusoh, M Paiman, AS Halim

**Affiliations:** 1 Reconstructive Sciences Unit, Universiti Sains Malaysia, Kubang Kerian, Malaysia; 2 Department of Orthopaedics, Universiti Sains Malaysia, Kubang Kerian, Malaysia

**Keywords:** vascular system injuries, lower extremity, rhabdomyolysis, renal dialysis, amputation

## Abstract

**Introduction:**

There is no consensus yet whether delayed limb salvage procedures with an “ischemic time” of more than 6 hours are worthwhile, as these repairs are often complicated by reperfusion injury. Our study aims to determine the outcomes of delayed revascularization to validate our current treatment approach and assess areas for future improvement.

**Material and Methods:**

We performed a retrospective cohort study on a highly selected group of patients who underwent delayed revascularization surgery for lower extremity traumatic vascular injuries in our institution from January 2008 to June 2018. Exclusion criteria include the presence of a terminal non-salvageable ischemic limb; defined as a gangrenous extremity evident by non-blanchable, mottled skin with complete limb paralysis, renal trauma, known renal impairment, and those with an ischemic time less than 6 hours. The demographic data, type and level of vascular, type of injuries, duration of ischemia, MESS score, and the need for secondary amputation were assessed. **Result:** Fifty-nine patients were identified and included in the analysis. Fifty patients (84.7%) were male, while 9 patients (15.3%) were female. The mean age was 28.1 years. The most injured vessel was the popliteal artery (n=41, 69.5%). The commonest injury pattern was contusion with thrombosis (n=31, 52.5%). Revascularizations were mainly achieved by interposition saphenous vein graft (n=40, 67.8%). The mean duration of delayed was 14.1 hours. A total of 83.1% of patients (n=49) had a Mangled Extremity Severity Scoring (MESS) of 7 and above. The limb salvage rate in this study was 89.8%, with only 6 patients (12.2%) requiring secondary amputations. Thirty-one patients developed rhabdomyolysis, with 6 cases (19.4%) requiring temporary inpatient renal replacement therapy (RRT). Out of the six, only one patient required lifelong RRT.

**Conclusion:**

Limb salvage in those with the duration of delayed of more than 6 hours should be attempted after careful assessment and a high rate of limb salvage, minimal renal complication and acceptable functional (mobility) outcomes can be achieved, despite the reperfusion injury that accompanies.

## Introduction

High-velocity trauma often causes severe skeletal and soft tissue injuries of the lower extremity. Vascular injury occurs in 4-6% of these cases, resulting in devastating and lasting disability^1,2^. In areas with limited access to health care, especially in developing countries, managing such injuries is challenging and controversial^3^. Malan and Tattoni introduced the concept of the 6-hour rule of revascularization^4^. However, there is still no consensus on limb salvage for those with a duration of ischemia of more than 6 hours. The merit of performing limb salvage in these patients is debatable due to the complications of reperfusion injury that follow^4^. Many authors have introduced various scoring systems to quantify the severity of the injury numerically and predict the viability of the injured limb, such as the Mangled Extremity Severity Score (MESS) introduced by Johansen *et al* in the 1990s^5-10^.

In developing countries, most cases are sent later than the golden period (within 6 hours after trauma) for revascularization due to a lack of efficient primary health care services and a limited number of equipped institutions to manage these cases^3^. Our institution is the regional referral centre for extensive extremity trauma with vascular injury. Due to logistical issues, most lower extremity traumas with vascular injuries referred to our institution have an ischemic time of more than 6 hours.

This study aims to determine the outcomes of delayed revascularization in terms of the need for secondary amputation, permanent renal impairment as a complication of reperfusion injury, and the functionality of the salvaged limbs in terms of mobility and ambulation— whether the patient can walk unaided, with walking aids, or with a wheelchair. We postulate that delayed revascularization can still yield satisfactory outcomes and a high limb salvage rate.

## Materials and Methods

A retrospective cohort study was performed on a select group of patients admitted to our institution from January 2008 to June 2018 who underwent delayed revascularization surgery for lower extremity traumatic vascular injury, with a duration from injury of more than 6 hours. Patients were identified through an electronic hospital operative database, and medical records were retrieved and reviewed. All patients with lower extremity vascular injuries confirmed by clinical examinations - such as absent distal pulses, absent or abnormal Doppler signals, and the presence of filling defects on computed tomography and angiography were included in this study. This included partially perfused distal extremities with good collateral circulation. Patients with a terminal, non-salvageable ischemic limb characterised by a gangrenous extremity with non-blanchable mottling and complete limb paralysis, renal trauma, known renal impairment, and those with an ischemic time of less than 6 hours were excluded. This study was designed according to the Strengthening the Reporting of Observational Studies in Epidemiology (STROBE) guidelines in line with the Declaration of Helsinki. Approval from the local ethics committee was also obtained (USM/JEPeM/18030182).

Epidemiological data, information on types and levels of vascular injury, vessels involved, types of vessel injuries, bony injuries, other associated injuries, duration of ischemia, the MESS score, and the need for secondary amputation were analysed. The MESS score is a scoring system applied to mangled extremities to predict and determine the need for salvage versus amputation after injury. It is a graduated grading system based on skeletal and soft tissue injury, shock, ischemia, and age^8,9^. A MESS of 7 or greater predicts the need for amputation^9^.

Survival of the injured limb is defined as the viability of the injured limb, evidenced by the presence of a distal pulse and good capillary refill time, with or without the presence of motor and sensory function. Duration of ischemia is defined as the time from the injury, as reported by first responders from the prehospital care ambulance service and medicolegally documented in the emergency department records, to the establishment of circulation post-unclamping following successful revascularization. Primary amputation is defined as the amputation of the injured limb without attempting salvage due to its extensive injury. In contrast, secondary amputation is carried out after a failed attempt at limb salvage. Renal function and creatinine kinase levels throughout the hospital stay were also recorded. All patients were followed-up for one year.

## Results

Sixty-eight patients were initially identified; however, only 59 patients were included in this study. Another 9 patients were excluded due to missing medical records and loss of follow-up. Fifty patients (84.7%) were male, while 9 patients (15.3%) were female. The mean age of the study population was 28.1 years (range 10 – 83 years). The majority of the patients were young adults aged between 18 and 30 years (n=25, 42.4%). Half of the patients (n=30, 50.8%) were motorcyclists involved in road traffic accidents with cars. Twenty-five patients (42.3%) sustained open fractures, 22 (37.7%) sustained closed fractures, 4 (6.8%) suffered knee dislocations, and 8 (13.2%) sustained only soft tissue and vascular injuries. Nine patients (15.3%) had other concurrent severe injuries.

**Fig. 1:**
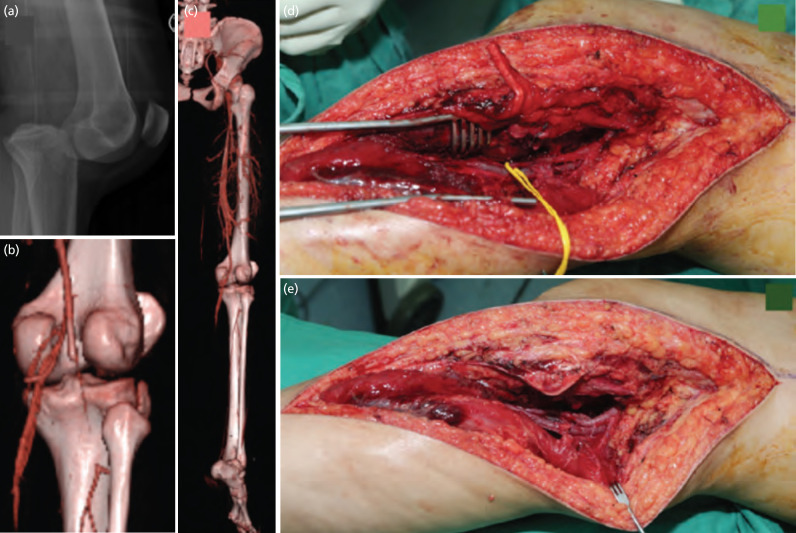
Case 1 – A 18-year-old motorcyclist sustained knee dislocation following head on collision with car. (a, b and c) CT angiogram showed 5cm segment of filling defect over popliteal artery with good distal runoff. (d and e) Intra-operative showed popliteal thrombosis and reverse saphenous vein graft was done for revisualisation. The posterior capsule and posterior cruciate ligament were reinforced with ethibond, and secondary stabilisation achieved with external fixation. The ischemic time was 10 hours due to delayed referral; he was ambulated with no distal neurology deficits. The posterior sagging was present with 0-90O range of motion otherwise the knee was stable for activity daily living.

**Fig. 2:**
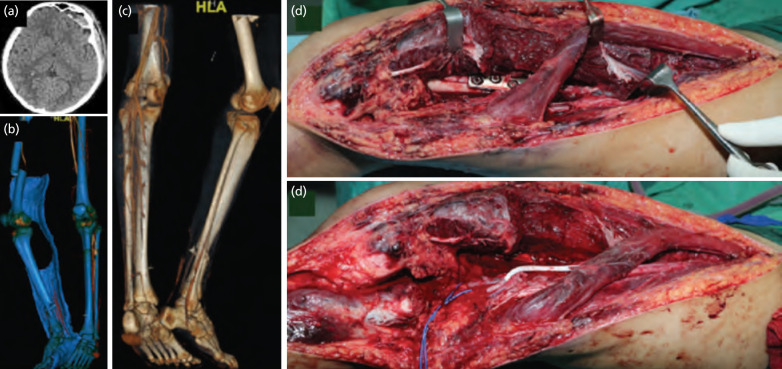
Case 2 – A 46-year-old man involved in road traffic accident whereby the thigh was rolled by other motorbikes. Trauma clearance revealed frontal contusion and multiple ribs fractures. (a) There is significant soft tissue contusion with fracture over midshaft femur and distal tibia. (b and c) CT angiogram showed complete filling defect over the fracture site with no distal run-off and collateral. (d and e) The circulation was established by PTFE graft 17 hour after injury however he developed renal impairments secondary to myoglobulinemia with high creatine kinase of more than 20,000 U/L. Secondary amputation was done at day 2 to minimise the systemic effect of reperfusion injury.

As shown in Table I, the most injured vessel in this series was the popliteal artery (n=41, 69.5%). The common pattern of injury was contusion with thrombosis (n=31, 52.5%), followed by complete transection (n=22, 37.3%). The mean length of vascular segmental loss or length of vessel required to be resected due to intimal injury was 6.0cm (range 0 – 20.0cm). Revascularizations with interposition saphenous vein grafts harvested from contralateral legs were required in 40 cases (67.8%), while 9 cases (15.3%) required a polytetrafluoroethylene (PTFE) graft due to the larger caliber of the injured vessels not amenable for saphenous vein graft reconstruction, as shown in Fig. 1 and 2, respectively. Open thrombectomy was performed in four cases (6.8%) where the length of thrombosis was less than 1cm. The mean duration of delayed was 14.1 ± 6.2 hours (range 6.0 to 37.0 hours).

**Table I:** Pattern of vascular injury and method of repair.

	**Vessels involve**				**Type of Injury**				**Method of Repair**				
	CFA	SFA	PA	PPTA	Complete transection	Partial transection	Thrombosis	Compression	Primary anastomoses	Reverse saphenous vein graft	PTFE	Open thrombectomy	Release
No (n)	2	11	41	5	22	1	31	5	2	40	9	4	4
Percentage(%)	3.4%	18.6%	69.5%	8.5%	37.3%	1.7%	52.5%	8.5%	3.4%	67.8%	15.3%	6.8%	6.8%

Abbreviations – CFA: common femoral artery, SFA: superficial femoral artery, PA: popliteal artery, PPTA: proximal posterior tibial artery, PTFE: polytetrafluoroethylene

In this study, 83.1% (n=49) had a MESS of 7 and above. Of these 49 cases, only 6 patients (12.2%) required secondary amputations, as illustrated in Table II. Thirty-one patients were critically ill with a mean peak creatinine kinase level of 11,686 U/L, suggestive of extensive rhabdomyolysis. Of these 31 cases, 6 cases (19.4%) required renal replacement therapy (RRT) due to a worsening renal profile with reduced urine output of less than 0.5 ml/kg/hr.

**Table II:** Cases required secondary amputation.

**Case no.**	**Injured vessel**	**Age (years)**	**Method of repair**	**Ischemic time (hours)**	**Bony injury (fracture)**	**MESS Score**	**Associated injuries**	**Indication of amputation**	**Delayed amputation (Days after vascular repair)**
1	Popliteal	20	vein graft	7.0	Open tibia MIC	11	Intracranial hemorrhage	insensate foot; osteomyelitis	148
2	Popliteal	46	PTFE graft	19.0	Closed femur	11	Rib fracture; cerebral concussion	extensive muscle necrosis	2
3	Femoral	22	vein graft	17.3	Closed femur	11	no	extensive muscle necrosis	4
4	Femoral	83	PTFE graft	7.4	Open femur and closed tibia	14	Cerebral concussion	hemodynamic unstable; coagulopathy, coagulopathy, repeated bleeding from vascular repair site	11
5	Popliteal	29	vein graft	27.5	Open tibia MIC	8	no	hemodynamic unstable; extensive muscle necrosis	7
6	Popliteal	22	vein graft	17.0	Open tibia MIC	10	no	sepsis with extensive muscle necrosis	181

Subsequently, two cases were discharged with renal impairment not requiring RRT, and one case of chronic diabetes with stage III chronic kidney disease required lifelong RRT. Unfortunately, out of these 6 cases requiring renal replacement therapy, one patient succumbed to his condition due to septicemic shock from infected wounds and complications from bowel perforation (Table III). The limb salvage rate in this series was 89.8%.

**Table III:** Cases required renal replacement therapy.

**Case no.**	**Injured vessel**	**Age (years)**	**Ischemic time (hours)**	**MESS score**	**Peak creatinine kinase level (U/L)**	**Peak creatinine level (umol/L)**	**Reason for renal replacement therapy**	**Renal function upon discharge**
1	Popliteal artery	21	15.5	H	42670	374	hyperkalemia, severe metabolic acidosis, oliguria, high CK	impairment not requiring RRT
2	Femoral artery	46	7.2	7	1918	669	hyperkalemia, oliguria	impairment requiring lifelong RRT
3	Popliteal artery	35	12.0	11	17695	264	hyperkalemia, metabolic acidosis, oliguria, high CK	Deceased
4	Femoral artery	36	11.3	11	22208	483	hyperkalemia, high CK,	no impairment
5	Femoral artery	83	7.4	14	37970	466	hyperkalemia, oliguria, high CK	impairment not requiring RRT
6	Popliteal artery	47	10.8	7	288831	163	high CK	no impairment

Abbreviations – CK: creatinine kinase, RRT: renal replacement therapy

The MESS score was higher in those cases requiring secondary amputation (7.9 vs 10.8, p<0.01) as shown in Table IV; nonetheless, the association of the MESS score with the viability of the limb after revascularization was insignificant [χ^2^(1) = 0.24]. It is worth noting that an association between limb ischemia and the shock factor of the MESS score with limb viability was still observed. Factors such as bony injury and associated injuries were associated with the limb's viability after revascularization surgery. The association between the peak level of creatinine kinase and the need for RRT was not statistically significant [χ^2^(1) = 0.33, p=0.57]. Those who underwent secondary amputation required longer hospital stays than those whose limb was successfully salvaged (33.1 vs 63.8 days, p=0.02).

**Table IV:** Multiple variable analysis for salvage and secondary amputation.

**Variables**	**Salvaged (n=53)**	**Secondary amputation (n=6)**	**P value**
	**Mean (SD)**	**Mean (SD)**	
Age (years)	27.13 (15.22)	37.00 (24.50)	0.16
Duration of ischemia (hours)	13.90 (6.00)	15.87 (7.73)	0.46
Length of vascular injury/thrombosis (cm)	5.65 (3.51)	8.80 (6.45)	0.09
MESS Score			
Limb ischemia score	4.79 (1.06)	6.00 (0.00)	0.01*
Patient age score	0.45 (0.67)	0.50 (0.84)	0.87
Shock score	0.26 (0.47)	1.00 (0.63)	0.00*
Injury mechanism score	2.53 (0.91)	3.33 (0.82)	0.04*
Total MESS score	7.91 (1.68)	10.83 (1.94)	0.00*
< 7	10	0	0.24
7 and above	43	6	
Duration of ischemia (hours)	6 – 12	27	2
0.41			
> 12	26	4	
Level of vascular injury	above knee	11	2
0.65			
knee level	38	4	
below knee	4	0	
Associated injuries			0.01*
Peak creatinine kinase level (U/L)	10707.68 (9445.84)	20819.00 (15142.83)	0.11
Renal impairment upon admission			0.43
Renal replacement therapy		0.58	

Upon follow-up in the clinic one year after discharge, 7 cases (14.8%) suffered delayed union of fractures; 2 (4.25%) had malunion, while another 7 (14.8%) suffered non-union, requiring further reconstruction. Ten patients (19.2%) required intermittent mild analgesia due to non-debilitating chronic pain. Twelve patients (20.4%) required crutches for ambulation, while 7 patients (11.9%) depended on wheelchairs. Patients' functional mobility was significantly associated (Pearson Chi-Square) with fracture healing status (p<0.001), limb ischemia/delayed >6 hours (p=0.022), and age. Also, bony union of fractures and factors limiting daily activities (pain, stiffness, etc.) were significant (p=0.007). The rest of the factors were not significant, including a MESS score of 7 and above, with a p-value of 0.06 (>0.05). No objective measurements of muscle and sensory function were available for analysis (Table V).

**Table V:** Mobility and walking aid used.

**Factors**			**Patient score (Age)**			**MESS score > 7**			**Facture**		
			**<30**	**30-50**	**>50**	**Yes**	**No**	**No Fracture**	**Union**	**Delayed Union**	**Non-Union**
Mobility and walking aid used	No aid	Count Expected count % within Mobility and walking aid used	26 21.6 78.8%	5 8.3 15.2%	2 3.2 6.1%	10 6.3 30.3%	23 26.7 69.7%	9 6.3 27.3%	21 16.5 63.6%	2 4.4 6.1%	1 4.4 3%
	Single crutch/ cane	Count Expected count % within Mobility and walking aid used	2 5.2 25%	4 2.0 50%	2 8 25%	0 1.5 0%	8 6.5 100%	0 1.5 0%	2 4 25%	4 1.1 50%	2 1.1 25%
	Two crutches/ canes	Count Expected count % within Mobility and walking aid used	4 2.6 100%	0 1.0 0%	0 4 0%	0 8 0%	4 3.2 100%	1 8 25%	2 2 50%	0 0.5 0%	1 0.5 25%
	Wheelchair	Count Expected count % within Mobility and walking aid used	2 4.6 28.6%	4 1.8 57.1%	1 7 14.3%	7 5.7 100%	7 5.7 100%	0 1.3 0%	1 3.5 14.3%	1 0.9 14.3%	3 0.9 42.9%
Total		Count Expected count % within Mobility and walking aid used	34 34 34 65.4%	13 13.0 13.0 25.0%	5 5.0 5.0 9.6%	10 10.0 10.0 19.2%	42 42.0 42.0 80.8%	10 10 10 19.2%	26 26 26 50%	7 7 7 13.5%	7 7 7 13.5%
		**Age**	**Value /df/Asymptotic significance Mess score**					**Fracture /Bony Union**			
Pearson Chi-Square		15.206a/6/0.019	7.128a/3/0.068					39.100a/12/<0.001			
Likelihood Ratio		15.857/6/0.015	10.428/3/0.015					34.790/12/<0.001			
Linear by linear Association		3.556/1/0.059	4.577/1/0.032					17.325/1/<0.001			
N of Valid cases		52	52					52			

## Discussion

Treatment of vascular injury in lower extremity trauma remains a topic of debate. It lacks a well-established algorithm, especially in cases that have surpassed the 6-hour "golden period." The goal of managing vascular injury in lower extremity trauma extends far beyond the viability of the salvaged limb. Multiple factors must be considered, such as the extent of bone and soft tissue injuries that will affect functional outcomes, the impact of reperfusion injury on the patient's general condition, and the duration of hospital stay, which might further impact the patient's psychosocial status.

The 6-hour "golden period" was initially coined based on an animal study by Miller and Welch *et al* in the late 1940s. The study revealed a 90% survivability of ischemic limbs after re-establishing circulation within 6 hours. Longer ischemic times resulted in a decrease in limb survival to as low as 20%^4,9^. Since then, many scoring systems have been devised to guide surgeons in deciding whether to salvage an injured limb, including the MESS score utilised in this study. Although a score equal to 7 or more is predictive of secondary amputation and such a limb should not be salvaged, with the advent of technology and surgical techniques, many authors have reported successful limb salvage even in cases with MESS scores of more than 711,12.

We excluded patients who fulfilled the criteria of having a terminal non-salvageable ischemic limb, also known as a dead limb, defined as a gangrenous extremity evident by non-blanchable mottled skin with complete limb paralysis^13^. Attempting to salvage such a limb is futile as the injury is irreversible and will lead to life-threatening reperfusion injury^14^. We only performed revascularization on viable injured limbs which had distal run-off on angiography, suggesting the presence of collateral vessels. The collateral supplies would have contributed to the viability of these limbs beyond the acceptable ischemic time. None of our patients required venous reconstruction as a total arteriovenous injury usually implies an extreme injury severing most vessels. This type of injury usually has no distal run-off on angiography, rapidly progressing to significant ischemia and subsequently falling into the group of dead limbs after 6 hours. All were excluded from revascularization and proceeded with primary amputation.

Patients with renal trauma or known renal impairment were also excluded due to the inherent risk of kidney injury posed secondary to reperfusion injury and rhabdomyolysis following limb revascularization. Primary amputation would have better benefited these patients to minimise morbidity and mortality rates. Although we did perform revascularization in those with an ischemic time of less than 6 hours, we did not incorporate this data as we did not intend to compare this subgroup, which has proven good clinical outcomes. A previous study done by our institution reported that 26.7% of vascular injuries that occur near our hospital underwent revascularization within 6 hours^15^.

All patients were managed with primary exploration and fasciotomy prior to revascularization. Due to the late presentation, fasciotomy is vital to minimise the risk of compartment syndrome and reperfusion injury. As all patients underwent open surgery, no endovascular intervention was undertaken. Upon exploration, we observed a varying degree of muscle reactivity upon stimulation and capability to bleed upon debridement. However, irrespective of these surgical findings, we did not analyse this subjective observation as it was not within the scope of this study.

We found no statistically significant association between the MESS score and the survival of limbs that underwent delayed revascularization. However, cases that required secondary amputation had higher MESS scores than those with successful limb salvage (10.83 vs. 7.91, p<0.01). Further evaluation revealed that the limb ischemia and shock components of the MESS score are associated with the limb's viability after revascularization surgery. Limbs requiring secondary amputation also had higher limb ischemia (6.00 vs. 4.79, p=0.01) and shock (1.00 vs. 0.26, p<0.01) scores. A combination of local ischemia and hemodynamic instability is a negative predictor for revascularization, as shock would have shut down peripheral circulation to maintain perfusion to centrally located vital organs. Additionally, those who underwent secondary amputation had more extensive bone (p=0.02) and other associated injuries (p=0.01). Upon one-year follow-up, 67.7% of patients were able to ambulate without aid and perform daily tasks.

Our study has demonstrated that dependency on the total MESS score is obsolete, and it should not be used alone in deciding whether salvage surgery should be performed. Although the MESS score needs thorough revision, we still favour the utilisation of the individual components of the MESS score instead of the total score, namely ischemia and shock status. One should also consider factors such as hemodynamic instability, extensive orthopaedic and crush injury, and other associated severe injuries such as intra-abdominal injury and traumatic brain injury.

It is also worth noting that our aim is not to promote delayed revascularization and undermine the importance of timely surgery. Any delay should be avoided at all costs if the situation permits. However, if the delay is inevitable, our decision on limb salvage should be scrutinised rather than taking a drastic measure of not salvaging the limb solely based on the ischemic time. We agree with Loja *et al* that decision-making should involve a multidisciplinary team approach with bedside patient evaluation^12^. This should include evaluating the patient's general condition (hemodynamic stability), the limb's state, and the surgeon's experience. Salvaging a non-functional limb will lead to more functional impairment than those primarily amputated and rehabilitated with a prosthesis^16^. On the other hand, primary amputation of a potentially salvageable limb will result in permanent disability for the patient^16^.

Reperfusion injury is one of the essential factors to be considered when managing lower extremity trauma with vascular injury. Severe reperfusion injury can lead to acute kidney injury (AKI). Muscle injury due to prolonged ischemia leads to rhabdomyolysis, causing the release of multiple substances such as myoglobin, iron, and free radicals. Myoglobin can precipitate in the kidney, causing renal tubular obstruction, while iron and free radicals cause oxidative injury and vasoconstriction-related hypoperfusion^17,18^. Creatine kinase (CK), an enzyme found in skeletal muscle, is released during muscular injury. It is detectable in serum and has been used as a marker for muscular tissue damage and ischemia. This is particularly useful in managing vascular injury in the lower extremities. It has been reported in the literature that a CK level of 5000 U/L or higher increases the risk of developing AKI. Some literature reported that a CK level of 1000 U/L was enough to cause a threefold increase in the risk of developing AKI. The current mainstay of therapy to prevent the development of AKI is the administration of intravenous fluid in the first 24 hours and targeting urine output at 200-300 ml/hr^17,18^.

According to the patient's weight, we tailored the intravenous fluids with a target urine output of 1-2 ml/kg/hour to achieve good hydration while avoiding fluid overload. A baseline renal profile and CK level were taken pre-operatively. Postoperatively, renal profile and CK level were taken at 12-hour intervals until CK level had fallen below 1000 U/L unless the patient developed AKI. Although all patients had a duration of ischemia of more than 6 hours, only 6 patients developed AKI requiring RRT. Upon discharge, only three patients had deranged renal profiles, and only one of them required lifelong RRT.

We advocate the current practice of early hydration and RRT for those at high risk of developing AKI. Additionally, patients with high creatine kinase with good urine output were managed with bicarbonate infusion to minimise the risk of kidney injury. Urinary alkalinization, as a direct effect of intravenous sodium bicarbonate administration, may reduce the pH-dependent generation of methemoglobin-containing tubular casts, ferrous-ion-catalysed production of free radicals, as well as oxidative damage related to proteinuria.

While the delay during inter-hospital transfer is beyond our control due to logistic and geographical circumstances, we strive to reduce the injury-to-intervention time by establishing a clear guideline in our emergency department (ED) to ensure initial assessment and referral to the respective teams can be done promptly. Additionally, our ED is located adjacent to the trauma operating theatres, well-equipped for microsurgery, and a 24-hour fully operational radiology department, further expediting the transfer for imaging and operation purposes. Unfortunately, no study has been undertaken to evaluate the efficacy of our pathway; hence, further study is warranted to re-evaluate our practice and identify potential resolvable issues. We highlighted two cases of delayed referral and associated injury that preclude early surgical intervention (Case 1 and 2).

We acknowledge some limitations identified in this study. Firstly, due to the retrospective nature of the review, some data were not available for analysis, including the objective measurement of muscle and sensory functions. Most clinical examinations only reported subjective assessments. As this was a single-centre study and only a highly select group of patients with viable limbs were recruited, the findings might be subject to bias and skewed towards more positive outcomes. There was no control group for comparison as well. Thirdly, we could not assess the long-term functional outcomes and complications due to our relatively short follow-up period. Although some success was reported after one year, long-term functionality or failure could not be verified. A future prospective study with a larger sample and a more extended follow-up period should be conducted to assess and validate additional contributing factors that may render salvage attempts unsuitable.

## Conclusion

Limb salvage in vascular injury with a delayed presentation of more than 6 hours should be attempted after a proper decision with a multidisciplinary approach, as observed in our study. However, we highlighted the risk of reperfusion injury and acute kidney injury following delayed revascularization. The MESS score alone should not be the deciding factor for salvage surgery; however, higher scores correlate with secondary amputation.
